# Severe skin and subcutaneous pythiosis in China: Metagenomic identification and characterization of *Pythium insidiosum*

**DOI:** 10.3389/fmicb.2022.1002460

**Published:** 2022-09-30

**Authors:** Haiyan Zhang, Fengli Zhou, Jiabao Huang, Xiaoyun Liu, Hui Xu, Jiayin Liang, Jun Wang, Jing Chen, Lingling Liu, Yiting Li, Xuan Hu, Xuanrong Chen, Chao Liu, Kouxing Zhang

**Affiliations:** ^1^Department of General Practice, Third Affiliated Hospital of Sun Yat-sen University, Guangzhou, China; ^2^Department of Clinical Laboratory, Third Affiliated Hospital of Sun Yat-sen University, Guangzhou, China; ^3^Hangzhou Matridx Biotechnology Co., Ltd, Hangzhou, China; ^4^Department of General Intensive Care Unit, Third Affiliated Hospital of Sun Yat-sen University, Guangzhou, China

**Keywords:** infection, metagenomic next-generation sequencing, pythiosis, *Pythium insidiosum*, trimethoprim-sulfamethoxazole

## Abstract

*Pythium insidiosum* is a rare fungus-like pathogen that is known to cause pythiosis in mammals with high morbidity and mortality. Identification of the pathogen is essential for timely treatment and rational use of antibiotics. However, *Pythium insidiosum* is difficult to detect *via* conventional microbiological tests. The current gold standard is polymerase chain reaction, which is lacking in most hospitals since human pythiosis is rare in China. In this study, we used metagenomic Next-Generation Sequencing and identified *Pythium insidiosum* in a 56-year-old Chinese male who was hospitalized due to severe edema in the right lower limb with scattered darkening indurations. The patient had a history of cirrhosis and occupational exposure to swamp water. Serological level of immune biomarkers indicated immunodeficiency, and Proteinase 3-Anti-Neutrophil Cytoplasmic Antibody was positive. Surgical incision of the lesions revealed radiating and reticular cutaneous ulcers. Microbial infections were suspected but conventional tests failed to discover the etiology. Empirical use of penicillin, vancomycin, and ceftriaxone had no effect. As a result, the peripheral blood and tissue biopsies were sent for metagenomic Next-Generation Sequencing, which reported *Pythium insidiosum*. This finding was corroborated by pathological staining, whole-genome sequencing, and internal transcribed spacer sequencing. Notably, antifungal treatment was ineffective, but the patient responded well to oral trimethoprim–sulfamethoxazole, which may be due to the *folp* gene found in *Pythium insidiosum* genome. Our study prompts future studies to determine the optimal treatment of skin pythiosis.

## Introduction

*Pythium insidiosum* (*P. insidiosum*) can cause pythiosis in mammals, which is an infectious disease with high morbidity and mortality (De Cock et al., 1987). Endemic to tropical regions such as Thailand, human pythiosis is classified into vascular, ocular, skin/soft tissue, and disseminated infections, while vascular and ocular infections are the most common clinical manifestation in humans (Krajaejun et al., 2006). Most regions in China belong to the subtropical zone and pythiosis is uncommon. Severe pythiosis affecting skin and soft tissues have not been previously reported.

Microbiological identification of *P. insidiosum* is essential for appropriate treatment and patient management. Conventional microbiological tests such as culture and smear tests are time-consuming and insensitive. For microbial cultures, sexual oogonia and hyphae-like morphological are the main identifying features of *P. insidiosum*, but similar morphologies can be seen in several fungal species. A reliable diagnostic approach is polymerase chain reaction (PCR) (Gaastra et al., 2010). However, PCR is a targeted method that requires *a priori* hypothesis and experiences of clinicians. Moreover, pythiosis is a rare disease and the relevant kits are lacking in most, if not all hospitals in China.

On the other hand, metagenomic Next-Generation Sequencing (mNGS) is a culture-free and hypothesis-free technique that can detect a wide array of potential pathogens directly from specimens (Wilson et al., 2014). Here we report the identification and characterization of *P. insidiosum* by mNGS as the first clinical case of human skin and subcutaneous pythiosis in southeastern China.

## Materials and methods

### Sample processing for mNGS and whole-genome sequencing

Both necrotic tissue and peripheral blood were used for mNGS testing. The peripheral blood was stored in cell-free nucleic acid preservation EDTA tube (Streck) during transportation. The sample was centrifuged at 1,600 *g* for 10 min and the supernatant was centrifuged again at 16,000 *g* for 10 min to collect plasma. Cell-free DNA (cfDNA) was extracted from plasma (Matridx #MD049). The tissue biopsy was surgically removed from the skin ulcer and stored in sterile Phosphate Buffered Saline. The biopsy was homogenized by vortex for 30 s and centrifuged at 12,000 rpm for 3 min and the supernatant was used for mNGS.

For whole-genome sequencing (WGS) experiments, the necrotic tissue incised from the skin ulcer was directly inoculated on the blood agar plate. The colony grew well and covered the 9-cm plate in 48 h. Agar blocks containing the colonies were removed and incubated on a new blood agar plate and the resulting colonies were collected and used for WGS.

### Culture of *Pythium insidiosum* for zoospore detection

The colonies from the blood agar plates were used to induce zoospores for the purpose of microbiologically confirming *P. insidiosum*. Agar blocks containing the colonies were incubated on 2% Sabouraud dextrose agar plate at 37°C, and the resulting new colonies were transferred into 2% Sabouraud dextrose broth and incubated at 37°C for 4 h. The broth was then switched to ultrapure water and incubated for 1 h. Grass leaves and induction solution were finally added after removing the ultrapure water and incubated at 37°C overnight. This process was based on previous studies (Vanittanakom et al., 2004; Gaastra et al., 2010). Zoospores were induced and observed in the water culture.

### Whole-genome sequencing

For DNA extraction, 25 ml of GP1 solution (CATB+TrisHCL+EDTA) was added into the colonies, and 500 μl of beta-Mercaptoethanol was added. The mixture was lysed at 65°C for 30 min and centrifuged at 12,000 rpm for 10 min. The supernatant was obtained and phenol/chloroform/isoamyl alcohol was added to remove impurities, and then centrifuged at 12,500 rpm for 8 min to obtain the supernatant. This step was repeated twice, isopropanol was added, and the mixture was incubated at −80°C for 20 min, and centrifuged at 12,000 rpm, 10°C for 10 min. The supernatant was discarded, and the precipitant was washed twice with 75% ethanol. Approximately 2 μg of genomic DNA was extracted and purified from the cultured colonies for WGS.

Sequencing data were generated using Illumina Novaseq 6,000 and PacBio sequel II, with a total coverage of approximately 84.61X (4.726 Gb) and 424.04X (23.68 Gb, N50: 15.561 kb), respectively. SMRT Link v5.0.1 was used to assemble the genome of Pacbio sequencing data. The preliminary assembly results were used as the reference genome, and bwa-0.7.8 software was used to align and correct the reads obtained by Illumina sequencing. The vcf file was generated *via* samtools v1.9 and bcftools v1.8. The final assembly was obtained using the corrected Pacbio sequencing results (quality score > Q20 and sequencing depth > 4X). After assembly with Pacbio long reads and Illumina PE150 reads data, 478 contigs with a total length 55.7 Mb of genomic sequences were completed. The genome assembly is of the best quality within all available *Pythium insidiosum* reference sequences and the GC content was 56.69%.

### Metagenomic sequencing

For DNA extraction, we used a kit from Matridx, Cat# MAR002, and followed SOPs provided by the manufacturer. Peripheral blood and tissue sample cut from the skin ulcer were sent for mNGS test. For peripheral blood, the sample was centrifuged at 16,000 *g* for 10 min and cell-free DNA was extracted from plasma.

The DNA underwent library preparation (enzymatic fragmentation of genomic DNA, end repairing, terminal adenylation, and adaptor ligation) and purification. All steps were performed according to previous publications (Luan et al., 2021; Zhou et al., 2022). For plasma cfDNA, no enzymatic fragmentation was included since cell-free DNA is already fragmented. Library concentration was quantified by real-time PCR (KAPA) and sequenced on Illumina Nextseq. Approximately 20 million of 75 bp single-end reads were generated for each library. Raw sequencing data were analyzed by the following bioinformatic pipeline: (1) trim of adapter sequences and low-quality bases (Q-score cutoff 20); (2) filtration of human-origin sequences *via* mapping to the human reference genome (GRCh38.p13) using BWA, which can be found at http://bio-bwa.sourceforge.net; and (3) alignment of the remaining reads after removal of low-complexity reads by BWA to a reference microbial database (NCBI nt and GenBank) to identify microbial species.

### Average nucleotide identity analysis

Average Nucleotide Identity (ANI) analysis was performed using a mixture of 17 published reference assembly sequences from the *Pythium* Genus in the NCBI database and was calculated using the Python (v3.7.8) module pyani (v0.2.10) based on MUMmer (ANIm) algorithms.

### Ethical approval and informed consent

The study was approved by the institutional research ethics committee of the Third Affiliated Hospital of Sun Yat-sen University, and written informed consent was obtained from the patient. The ethics committee reference number is [2021]-02-137-01.

## Results

### Case presentation

In December 2020, a 56-year-old Chinese male was admitted due to multiple skin ulcers in the right leg. Prior to hospitalization, the patient had hepatitis B-induced cirrhosis and splenic artery embolization for the treatment of hypersplenism. The patient had direct contact with swamp water due to his occupation as a farmer and therefore infectious diseases were considered. However, conventional microbiological tests reported no findings.

The serological level of immune markers suggested that the patient was immunodeficient (serum complement-3 0.5g/L, serum complement-4 0.04g/L). Flow cytometry of peripheral blood cells showed a CD4+ T lymphocyte count of 156/ml, CD8+ T lymphocyte count of 88/mL, and CD3 + T lymphocyte count of 264/mL. Following empirical antibiotic treatment of vancomycin for 10 days and teicoplanin for 5 days, the patient had voluntarily left the hospital with only mild improvement.

In February 2021, the patient was hospitalized again due to severe swelling of the right lower limb. Non-pitting edema with scattered darkening indurations was observed and skin temperature of the indurated area was elevated. The level of serum inflammatory markers was slightly elevated (procalcitonin 0.114 ng/mL, C-reactive protein 23 mg/L). Blood tests reported reduced level of blood cells (white blood cell 1.91 × 10^9^/L, neutrophil 1.26 × 10^9^/L, lymphocyte 0.36 × 10^9^/L, eosinophil 0.18 × 10^9^/L, hemoglobin 70 g/L, platelet 74 × 10^9^/L). Coagulation markers reported prothrombin time of 18.8 s, fibrinogen of 2.48 g/L, and activated partial thromboplastin time of 43.8 s. Liver function test showed slightly increased transaminase and significantly decreased albumin (17 g/L). Immune function test indicated immunodeficiency (CD4+ T lymphocyte 192/mL, CD8+ T lymphocyte 168/mL, CD3+ T lymphocyte 380/mL, serum C3 0.81 g/L, serum C4 0.08 g/L). Proteinase 3-Anti-Neutrophil Cytoplasmic Antibody (ANCA) was 92, and cytoplasmic-ANCA was weakly positive. The filarial antibody was negative. No microfilariae were found in peripheral blood smear. Color ultrasound and Computed Tomography Angiography (CTA) of the lower extremities showed no deep venous thrombosis and arterial embolism.

Based on the recurrent lesion, abnormal immune biomarkers, and ANCA test results, clinicians initially considered the possibility of ANCA-associated vasculitis. The patient was administered 40 mg methylprednisolone daily. However, the patient’s lower limb exhibited progressive swelling and rupture of indurations at day 15 after admission. Multidisciplinary consultation suggested the likelihood of erysipelas, but penicillin, vancomycin, and ceftriaxone treatment had no effect. Rather, several deteriorated radiating and reticular subcutaneous ulcers formed. Finally, the clinicians performed surgical incisions of the ulcerous lesions and sent the biopsy for microbiological, pathological, and mNGS testing.

### Identification of *Pythium insidiosum via* mNGS and WGS

**Figure 1:**
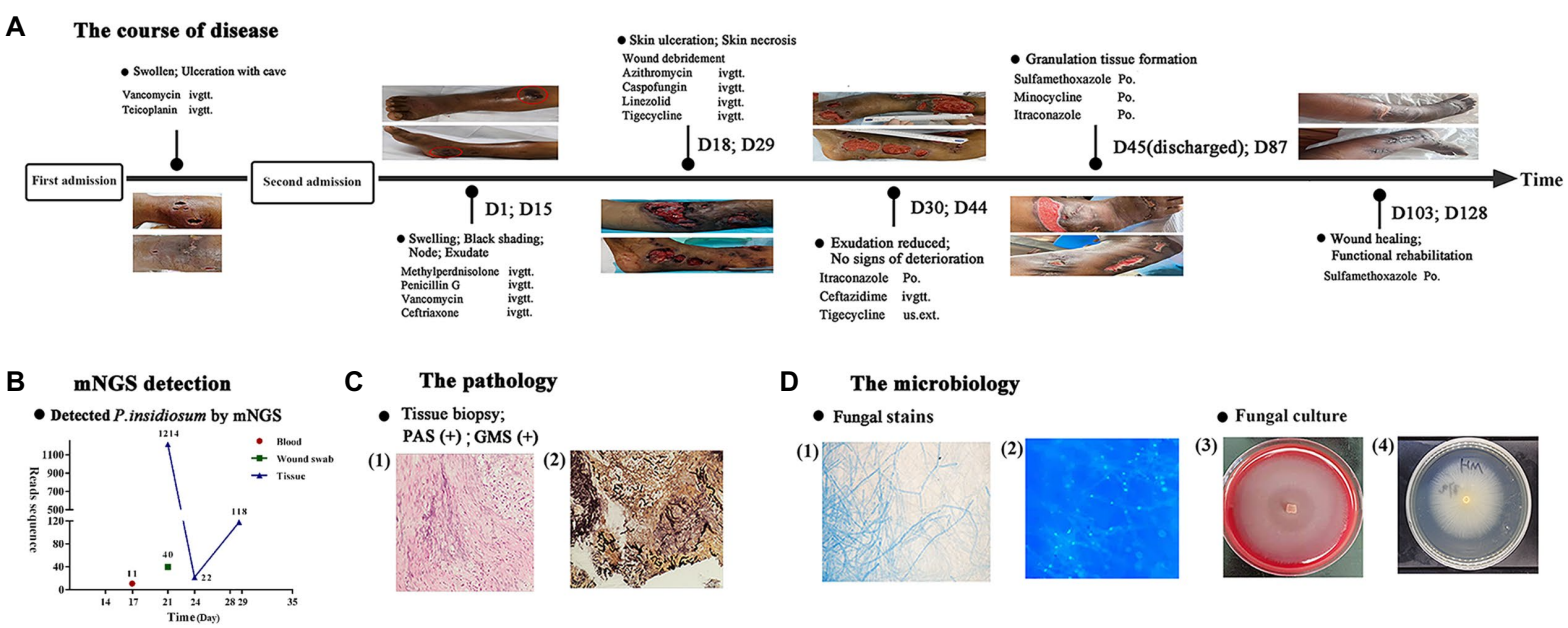
Flowchart of the diagnosis and treatment of the patient. **(A)** The course of disease over time. **(B)** The sequencing reads of mNGS. **(C)** Histopathology of the skin ulcer: (1) periodic acid-Schiff staining, (2) Gomori methenamine silver staining. **(D)** Staining of cultured hyphae: (1) gram staining, (2) immunofluorescence staining and images of fungal culture: (3) blood agar medium, (4) potato dextrose agar medium.

**Figure 2:**
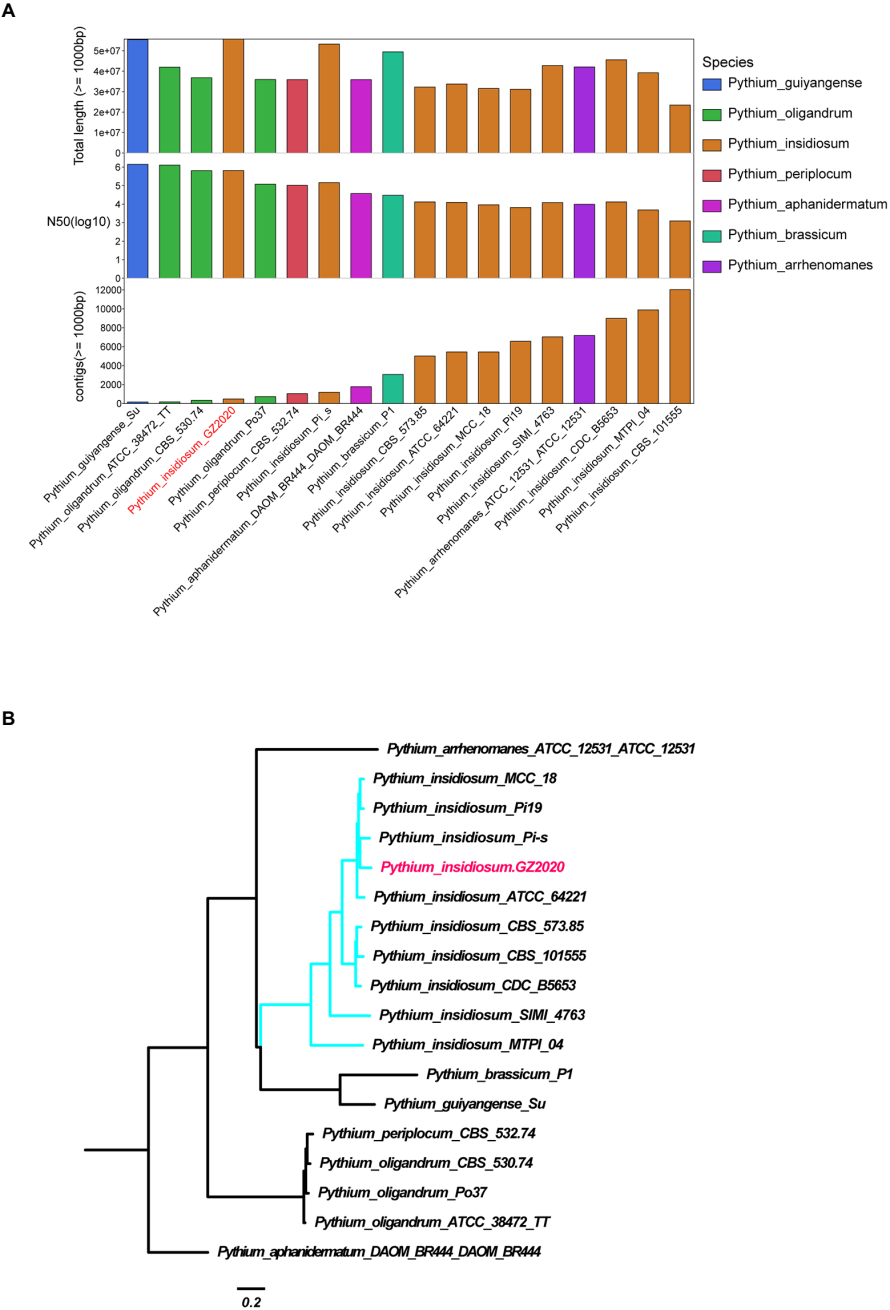
Identification of Pythium insidiosum. **(A)** Comparison of the assembly quality of Pythium strains reported on NCBI and GZ2020. Longer total length, N50, and lower number of contigs reflect better assembly. **(B)** Genome-wide SNP system tree. Mummer software was used to conduct SNP calling (Pythium aphanidermatum as reference strain) and to construct phylogenetic tree using all SNP sites.

The peripheral blood of the patient was sent for mNGS on day 16 after admission. On day 17, plasma mNGS reported that 73.3% of fungal reads aligned to *P. insidiosum* (11 out of 15 reads, Figure 1). Subsequently, a biopsy taken from skin ulcers was sent for mNGS to validate the plasma results, which again identified 1,214 reads of *P. insidiosum*. On day 26, the colony incubated from tissue biopsy was sent for whole-genome sequencing, and more than half of 478 contigs shared a 97.36% similarity with the reference genome of *P. insidiosum* strain Pi-s (GCA_001029375.1) (Figure 2).

### Confirmatory tests for *Pythium insidiosum*

The swabs, biopsy, and exudation samples were sent for conventional microbiological tests. On day 18, the smear test showed septate hyphae. Histopathological examination of the biopsy revealed inflammatory granulation tissue with eosinophil infiltration. Both Periodic acid-Schiff (PAS) stain and Gomori methenamine silver (GMS) stain showed fungal hyphae and spores, indicating the presence of potential fungal-like pathogens. To further identify the pathogen, a tissue biopsy was sent for culture, and the zoospores of *P. insidiosum* were induced in water culture with grass leaves and induction solution. In addition, using DNA extracted from the biopsy, PCR was performed to amplify the internal transcribed spacer (ITS) region and Sanger sequencing results were aligned to the NCBI NT database using the Blast program. Among 107 valid results, 104 of which were identified as *P. insidiosum*.

### Antimicrobial treatment and outcome

The patient was administered 40 mg methylprednisolone daily for 10 days. However, the patient’s lower limb exhibited progressive swelling and rupture of indurations at day 15 after admission. Multidisciplinary consultation suggested the likelihood of erysipelas, but penicillin (12 million unit per day, for 5 days), vancomycin (1.0 g, every 12 h, for 6 days), and ceftriaxone (2.0 g/day for 6 days) treatment had no effect. Rather, several deteriorated radiating and reticular subcutaneous ulcers formed.

From days 18 to 29, venous azithromycin (0.5 g/day for 1 week), caspofungin (first dose 70 mg/day, followed by 50 mg/day for 20 days), and linezolid (600 mg, every 12 h, for 2 weeks) were administered. One week later, azithromycin was adjusted to tigecycline (50 mg, every 12 h, for 10 days). No new lesions were seen, and the exudation of skin ulcer was significantly reduced on day 30. Considering the potential risk of linezolid-induced thrombocytopenia and indigestion associated with tigecycline, as well as the financial constraints of the patient, the therapy was changed to oral itraconazole (0.2/day, for 5 days), intravenous ceftazidime-tazobactam (1.2 g, every 8 h, for 4 days), and topical tigecycline. The patient was discharged on day 45 with prescriptions of oral trimethoprim–sulfamethoxazole (TMP-SMZ) twice a day (2 tablets 160/800 mg, each tablet contained 80 mg trimethoprim and 400 mg sulfamethoxazole), minocycline (100 mg/day), and itraconazole (0.2/day).

Multiple follow-up visits were conducted. On day 87, small ulcers have healed, and shrinkage of larger ulcers was seen. On day 103, the antibiotics were de-escalated to oral TMP-SMZ only. On day 128, most skin ulcers have healed (Figure 1). In the latest follow-up visit on 10th January 2022, all skin lesions have successfully healed. However, due to the patient’s underlying condition of hypersplenism, the white blood cell and neutrophil count were still lower than normal (3.06 × 10^9^/L and 1.75 × 10^9^/L, respectively).

## Discussion

Pythiosis is a life-threatening condition. It typically occurs through exposure to zoospore-contaminated water (Krajaejun et al., 2006). In our case, the patient may be affected during exposure to swamp water. The patient showed a rapid deterioration after the onset of symptoms, possibly due to the underlying conditions including cirrhosis, neutropenia, anemia, and glucocorticoid therapy. Skin pythiosis has been reported to induce chronic wound, cellulitis, chronic ulcers, or infiltrative mass akin to tumor (Triscott et al., 1993; Bosco Sde et al., 2005). However, swelling at the site of lesion is uncommon, and few studies have reported severe soft-tissue infection at an early stage of pythiosis. Moreover, *P. insidiosum* is difficult to detect *via* traditional microbiological tests. A reliable diagnostic approach is PCR (Gaastra et al., 2010). However, PCR is a targeted method that requires hypothesis and prior experiences of clinicians. In addition, pythiosis is a rare disease and the relevant kits are lacking in most, if not all hospitals in China. According to a study that reported 102 cases of human pythiosis, the duration of symptoms can last 3 months without correct diagnosis and treatment (Krajaejun et al., 2006). In our case, the atypical symptoms and negative results of conventional tests preclude an early diagnosis of the patient, and metagenomic sequencing provided essential clues on day 17 after admission.

The main advantage of mNGS was that it could detect almost all pathogens with known genomic sequences in a single test. Other techniques such as ITS and 16S rRNA are also great clinical diagnostic tools, but they can only cover a limited range of potential pathogens. Therefore, we elected to use mNGS, which is widely available as a “send-out” test in many hospitals in China. On the other hand, mNGS has limitations such as its high cost so not all patients are recommended to use this technique. We only suggested using mNGS on febrile patients in severe conditions awaiting timely diagnosis when conventional microbiological tests were negative.

The treatment of pythiosis in our case was of interest. *Pythium insidiosum* was usually treated by antifungals (Agarwal et al., 2018). However, there was a high rate of treatment failure, which might be due to the lack of ergosterol (the molecular target of antifungal agents) on the organism’s cell membrane (Schlosser and Gottlieb, 1966). There were cases that were successfully treated with amphotericin B (Triscott et al., 1993), itraconazole, and terbinafine (Shenep et al., 1998; Argenta et al., 2008). And *in vitro* study showed that *P. insidiosum* was susceptible to caspofungin (Abuhammour and Habte-Gaber, 2004). However, in our case, neither caspofungin nor itraconazole was effective. Recent studies have suggested that antibiotics may work better than antifungals to treat pythiosis (Loreto et al., 2014), and both *in vitro* and *in vivo* studies have been conducted to analyze the effect of azithromycin, clarithromycin, minocycline, and tigecycline. The result indicated that a combined use of these antibiotics showed synergistic effects in treating pythiosis (Jesus et al., 2016). In our case, there was only mild improvement using linezolid and tigecycline. Rather, TMP-SMZ worked well, resulting in significant resolution of symptoms. We discovered the *folP* gene in *P. insidiosum* genome encoding dihydropteroate synthase (DHPS), which is the main target of Sulfonamides and could partially explain the satisfying effect of TMP-SMZ (Griffith et al., 2018). Nevertheless, our case prompts future studies to determine the optimal treatment of pythiosis in immunodeficient patients.

## Data availability statement

The datasets presented in this study can be found in online repositories. The names of the repository/repositories and accession number(s) can be found in the article/supplementary material. The original fastq data supporting the conclusion of this article is available in the [NCBI] data base with accession number [PRJNA808783] and hyperlink to the database: [https://www.ncbi.nlm.nih.gov/bioproject/PRJNA808783].

## Ethics statement

The study was approved by the institutional research ethics committee of the Third Affiliated Hospital of Sun Yat-sen University, and written informed consent was obtained from the patient. The ethics committee reference number is [2021]-02–137-01. The patients/participants provided their written informed consent to participate in this study.

## Author contributions

HZ, FZ, and KZ designed the study and collected the data. JH, XL, HX, JL, LL, YL, XH, and XC provided and analyzed clinical data. JW, JC, and CL provided and analyzed metagenomic next-generation sequencing data and wrote the draft. All authors contributed to the article and approved the submitted version.

## Conflict of interest

JC, JW, and CL are employees of Matridx Biotechnology Co., Ltd.

The remaining authors declare that the research was conducted in the absence of any commercial or financial relationships that could be construed as a potential conflict of interest.

## Publisher’s note

All claims expressed in this article are solely those of the authors and do not necessarily represent those of their affiliated organizations, or those of the publisher, the editors and the reviewers. Any product that may be evaluated in this article, or claim that may be made by its manufacturer, is not guaranteed or endorsed by the publisher.
